# Statewide Intensification of Harmful Algal Blooms Across California Lakes and Reservoirs

**DOI:** 10.1029/2025GH001627

**Published:** 2026-04-03

**Authors:** Brittany N. Barreto Martinez, Erin L. Hestir, Marc W. Beutel, Christine M. Lee

**Affiliations:** ^1^ Department of Civil, Construction, and Environmental Engineering San Diego State University San Diego CA USA; ^2^ Department of Civil & Environmental Engineering University of California Merced Merced CA USA; ^3^ NASA Jet Propulsion Laboratory California Institute of Technology Pasadena CA USA

**Keywords:** satellite remote sensing, algal blooms, cyanobacteria, public health, water quality

## Abstract

Monitoring cyanobacteria is crucial for assessing water quality, safeguarding public health, and understanding ecosystem dynamics impacted by harmful algal blooms. This study explores the potential of satellite remote sensing (SRS) to assess risks of cyanotoxin exposure in California's recreational waters from 2002 to 2011 and 2016 to 2023. Utilizing SRS data, we compared cyanobacteria abundance across five lakes with cyanotoxin data and advisories from the California Department of Water Resources (DWR). SRS‐based advisories were aligned with DWR/in situ based advisories 54%–100% of the time. Lake‐specific assessments of agreement showed Lake Oroville with the highest overall accuracy (100%) and Pyramid Lake with the lowest (54%). SRS generally overpredicted DWR‐based alerts in about 30% of instances and under‐detected DWR‐based alerts at a rate of 42%, likely due to differences in the way satellites sample across continuous spatial domains but at coarse resolutions versus in situ sampling at discrete locations. We extended our SRS monitoring capability to an additional 71 lakes to conduct a statewide assessment of toxin alerts over time. There were 10 lakes that experienced cyanobacteria alerts 12%–88% of the time across our study. When comparing 2002 to 2011 and 2016 to 2023, we observed higher rates of toxin alert frequency, duration, and a shift toward earlier starts of the year for high‐risk blooms across all regions of California, with the greatest in southern California. Despite limitations in spatial resolution, SRS provides consistent, near‐real‐time data essential for timely cyanotoxin risk assessments and public health alerts, complementing traditional in situ sampling.

## Plain Language Summary

This study investigates the potential of satellite remote sensing (SRS) to assess risks of cyanotoxin exposure in California's recreational waters from 2002 to 2011 and 2016 to 2023. We validated the SRS‐derived cyanobacteria biomass estimates against concurrent field measurements from the California Department of Water Resources (DWR) across five key California lakes, demonstrating strong statistical agreement. Subsequently, we utilized the validated SRS data to evaluate high‐risk bloom frequency and duration against established public health thresholds for potential cyanotoxin exposure. Our analysis revealed satellite‐based warnings agreed with DWR's alerts between 54% and 100% of the time, with Lake Oroville showing the best agreement and Pyramid Lake the lowest. While satellites sometimes predicted alerts when DWR did not (∼30%) or missed them when DWR issued one (∼42%), this is likely due to the different ways they collect data. We expanded to 71 other lakes, where 10 had high‐risk cyanobacteria alerts 12%–88% of the time. When comparing both periods, we observed higher rates of toxin alert frequency, duration, and an earlier onset of high‐risk blooms across all regions, with the greatest seen in southern California. Despite some differences, satellite data offers a powerful tool for quickly and consistently assessing cyanotoxin risks.

## Key Points

- Satellite remote sensing (SRS) shows promising agreement with in situ methods for detecting harmful algal blooms in recreational waters
- The 2016 to 2023 analysis revealed an increase in high‐risk bloom frequency and duration across California, alongside an earlier onset
- Despite spatial resolution limitations, SRS offers a valuable, consistent, and near‐real‐time data source for cyanotoxin risk assessment

## 1 Introduction

Freshwater cyanobacteria occur naturally and play vital ecological roles as primary producers, nitrogen fixers, carbon sinks, and sources of oxygen (Bhardwag et al., 2024; Demoulin et al., 2019). Certain cyanobacteria species produce cyanotoxins, such as Microcystins, Anatoxins, Cylindrospermopsin, and Saxitoxins, which can pose risks to human health, animals, and the environment (Carmichael et al., 2001; Metcalf et al., 2021; Smayda, 1997; Turner et al., 1990). Cyanobacteria‐dominated harmful algal blooms (cyanoHABs) can lead to overabundant biomass and toxin production, adversely affecting recreation, drinking water consumption, livestock watering, fisheries, and irrigation use (Backer et al., 2013; Briand et al., 2003; Falconer & Humpage, 2005; Landberg, 2002; Stewart et al., 2006). Additionally, cyanoHABs create economic burdens such as increased water treatment expenses, loss of water access, healthcare costs, and diminished recreational opportunities (Anderson et al., 2000; Bingham et al., 2015; Graham et al., 2016; Kouakou & Poder, 2019). CyanoHABs are a worldwide phenomenon (Brooks et al., 2016; Intergovernmental Panel on Climate Change, 2022; Paerl & Huisman, 2009) and the frequency and intensity of cyanoHABs are a concern for many water managers and communities because of eutrophication and global warming (Meerhoff et al., 2022; Moss et al., 2011). Consistent monitoring of on‐site cyanoHABs allows for timely public health advisories and beach closures, directly reducing the risk of exposure to harmful toxins and safeguarding public safety.

In the United States the spatial coverage of water quality monitoring is limited (Schaeffer et al., 2018). Of the limited number of water bodies that are sampled for water quality, 90% have less than five sampling stations, and ∼50% have only one sampling station (Schaeffer et al., 2018). Satellite remote sensing (SRS) data can complement traditional in situ water quality monitoring for cyanotoxins by providing additional temporal and spatial coverage of cyanobacteria (Schaeffer et al., 2022; Urquhart et al., 2017; Wynne & Stumpf, 2015), thereby enhancing current monitoring programs (Papenfus et al., 2020; Schaeffer et al., 2015; Stroming et al., 2020). SRS cannot directly measure cyanotoxins (Stumpf et al., 2016). However, it has been used to identify areas where cyanotoxin concentrations are likely to be elevated based on the presence and density of cyanobacterial blooms (Kutser, 2009; Lunneta et al., 2015; Mishra et al., 2019).

California water bodies may have some of the highest Microcystin levels in the world, where in some cases it was >10,000 μg/L during bloom seasons (California Regional Water Quality Control Board, 2023). Nutrient runoff from agricultural fields and urban areas, coupled with its warm, semi‐arid climate, create ideal conditions for algal growth, making it the ideal state to focus on cyanoHAB monitoring. With California's diverse ecosystems, the findings and monitoring strategies described in this study could be applied elsewhere and at local, regional and even global scales.

The Department of Water Resources (DWR) manages California's water resources, systems, and infrastructure, including the State Water Project (SWP). The SWP is a vast, multi‐purpose system of canals, pipelines, reservoirs and hydroelectric facilities that delivers drinking water to 27 million Californians, irrigates over 300,000 ha of farmland, and supports businesses within the state (California DWR, 2024b). The DWR tests for cyanotoxins in their managed waters every year, however their testing is not year‐round and has limited sampling locations due to cost and time constraints. In instances where cyanotoxins are detected within SWP water bodies, the DWR advises recreational users to exercise caution and refrain from any direct contact with algae (DWR, 2024). Recreation may be restricted as a precautionary measure to safeguard public health if the algae are confirmed to be harmful. The DWR's recreational health advisory levels are based on a risk assessment conducted by the Office of Environmental Health Hazard Assessment (OEHHA), applying the best available science and a precautionary approach to public health (California CyanoHAB Network, 2016). Recently, Lopez Barreto et al. (2024) classified cyanobacteria abundance derived from SRS Sentinel‐3 sensor according to the World Health Organization's 1999 guideline values (WHO99 GV) for cyanobacteria in recreational waters. They compared the SRS guideline values against cyanotoxin public health advisories issued by the DWR for San Luis Reservoir, a keystone reservoir in the SWP. They found an 83% agreement between SRS and DWR advisories.

This study evaluates the applicability of SRS to assess large scale regional risk of public recreational exposure to cyanotoxins, extending the work of Lopez Barreto et al. (2024) to all waterbodies in SWP monitored by the DWR. This study tested the agreement between SRS and DWR advisories in an additional five lakes owned, managed, and monitored by the DWR, and tested the extensibility of the approach to a lake‐level assessment. In the previous Lopez Barreto et al. (2024) study, the cyanobacteria value used for comparison was from a pixel that was closest to the DWR sample collection site from their lake field sampling. However, using a single point to summarize the entire lake's cyanotoxin level potential is not leveraging the spatial capability of SRS. Here, we used SRS to summarize and classify cyanobacteria levels across five SWP lakes (San Luis, Castaic, Oroville, Perris, Pyramid) by WHO99GV, a departure from previous single‐pixel comparisons. We hypothesize that point‐based SRS data, particularly near DWR sample collection sites, will align better with DWR alerts than lake‐wide summaries, strengthening the case for SRS in public health warnings.

Our study also represents a significant extension of prior work by Urquhart et al. (2017) and other cyanoHAB studies (Coffer et al., 2021; Mishra et al., 2021; Schaeffer et al., 2022; Whitman et al., 2022) through the creation of a comprehensive analysis of high‐risk cyanobacterial bloom dynamics across 76 resolvable California lakes and reservoirs. This analysis utilizes the full available SRS data record from Envisat's MEdium Resolution Imaging Spectrometer (MERIS) and Sentinel‐3's Ocean Land Color Imager (OLCI) for the two periods 2002 to 2011 and 2016 to 2023.

Our study's novel contribution is the comparison of these satellite‐derived cell counts against in situ cyanotoxin data for five lakes. Specifically, we relate the satellite estimations of cyanobacteria abundance to the actual documented risk level (e.g., caution, warning, or danger advisories) as defined by the presence of specific toxins (such as Microcystin). For high‐risk lakes, we quantified the average number, start/end dates, and duration of these events based on the WHO99 GV, allowing us to identify the ten highest‐risk lakes and reservoirs for public recreational exposure to cyanotoxins. By determining metrics such as duration, persistence, and when the onset of high‐risk blooms occurs, our work moves beyond only bloom detection and frequency mapping to provide a direct, time‐series assessment of cyanotoxin risk across the state.

## 2 Methods

### 2.1 Study Site

**Figure 1:**
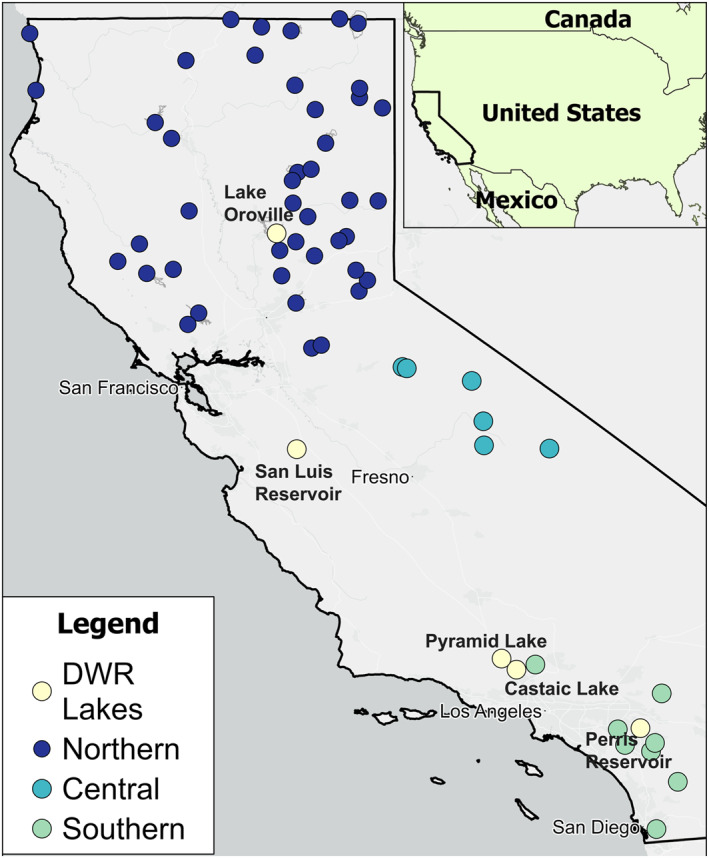
Map of the five State Water Project (SWP) reservoir sites (names in bold) across California, USA. Reservoir sizes are exaggerated for visualization purposes. All other lakes resolvable by SRS OLCI/MERIS are color labeled by region (northern, central and southern).

California encompasses a wide range of geographic and climatic conditions, including coasts, mountains, and arid landscapes (Bedsworth et al., 2018). Figure 1 depicts the five DWR‐operated reservoirs, and the other lakes used in our study. Northern California, which includes Lake Oroville typically features a climate with mild, wet winters and warm, dry summers (Ackerly et al., 2018; Houlton & Lund, 2018). In contrast, the San Joaquin Valley, hosting San Luis Reservoir, experiences hotter summers and milder winters due to its inland location, often exceeding 90°F (32°C) in summer months (Fernandez‐Bou et al., 2022). Southern California (Castaic Lake and Pyramid Lake) tends to be drier than its northern counterpart, with hot summers and mild, wet winters (Hall et al., 2018; Kalansky et al., 2018). Inland southern California (Perris Reservoir) tends toward a semi‐arid or desert climate, characterized by hot summers often surpassing 100°F (38°C) and relatively mild winters (Hopkins, 2018).

Our study sites include five reservoirs of varying size and trophic status, all of which are part of California's SWP. While the primary purposes of these reservoirs are for water supply, flood risk reduction or hydroelectricity (United States Army Corps of Engineers, 2024), all the reservoirs are commonly used for recreational activities, attracting residents and visitors for fishing, boating, swimming, hiking, and camping (California State Parks, 2024). More information regarding these SWP reservoirs can be found in Tables S1 and S2 of Supporting Information S1.

### 2.2 Cyanotoxin Validation Data

The SWP is a crucial water management system that plays a vital role in supplying water to California's urban and agricultural areas, supporting economic development, and ensuring environmental sustainability (California DWR, 2024b; Lund et al., 2010; United States Bureau of Reclamation, 2023). The primary purpose of the SWP is to transfer water from northern regions of the state to water‐deficient central and southern regions of California. Since 1968 the Division of Operations and Maintenance (O&M), the Environmental Assessment Branch of the DWR, has overseen water quality monitoring for the SWP. They initiated cyanotoxin monitoring around 2006 in key reservoirs in the SWP (California Department of Water Resources, 2024b).

The DWR samples for cyanotoxins about weekly beginning in mid‐April (spring) until late September/early October (late summer/early fall), which is the typical algal bloom season in California (California DWR, 2024a). They will sample for cyanotoxins prior to the expected bloom season if there are algal bloom sightings by local rangers or if there is a report made by the public. If a test is positive for cyanotoxins, the DWR will post corresponding health advisories at the sampling facilities, reservoir, and park entrances, and online (Table 1, DWR, 2024). Their routine weekly sampling will continue until toxin levels are below caution levels for two consecutive weeks. The DWR collects water samples at the surface from the shore or docks, 1‐m depth and raw water tap samples from lower or upper intakes, depending on the site. Toxins are measured using laboratory assay conducted by a DWR subcontractor, Greenwater Laboratories using ADDA‐ELISA kits. The DWR provides a fixed geo‐location of sampling sites with their reporting, but the actual position of the sample may vary based on water level. A detailed description of how these estimate cyanotoxins can be found in Lopez Barreto et al. (2024) or by contacting the DWR's O & M Environmental Assessment Branch.

**Table 1:** Guideline Values (GVs) for Recreational Waters by the WHO for Cyanobacteria and Advisories Levels Set by the CA Department of Water Resources (DWR) for Cyanotoxins

Authority	Authority guideline level	Value	Classification for this study
California Department of Water Resources Recreational Cyanotoxin Advisory Levels	Caution	0.8–5.99 μg/L Microcystins	No Alert
	Warning	6–19.99 μg/L Microcystins	Alert
	Danger	20 μg/L ≤ Microcystins	Alert
World Health Organization 1999 Guideline Values for Cyanobacteria in Freshwater	Relatively low probability of adverse health effects	≤20,000 cyanobacterial cells/ml or ≤10 chl‐a μg/L	No Alert
	Moderate probability of adverse health effects	100,000 cyanobacterial cells/ml or 10.1–50 chl‐a μg/L	Alert
	High probability of adverse health effects	≥100,000 cyanobacterial cells/ml or ≥50 chl‐a μg/L	Alert

For this study, we used only surface and 1‐m deep water samples to compare against SRS data. We used DWR data from 2016 to 2023 because while there was earlier DWR data available, sampling was very sparse. The DWR routinely screens for multiple toxins, including Microcystin, Anatoxin, and Saxitoxin. Microcystin was the dominant toxin at the five study lakes, with other cyanotoxins consistently remaining below the State of California's alert thresholds throughout the study period, which is why this study focuses on Microcsytin exclusively for all lakes.

### 2.3 Cyanobacteria Cell Counts From MERIS and OLCI

Cyanobacteria SRS products were obtained from CyAN version 5 (May 2023), a collective project between several federal agencies (CyAN, 2025). The cyanobacteria data, CI_cyano_ (CyAN, 2025; Schaeffer et al., 2015), is based on the modified Cyanobacteria Index (CI) (Lunetta et al., 2015; Wynne et al., 2008). The CI_cyano_ data for 2002 to 2011 are from the European Space Agency's (ESA) Envisat's MERIS and data from 2016 until present are from the ESA's Sentinel‐3 OLCI. MERIS operated on the Envisat satellite from April 2002 to April 2012, providing 300‐m spatial resolution imagery every 2–3 days. The sensor included 15 spectral bands (390–1,040 nm), with dedicated narrow bands in the red and near‐infrared region optimized for detecting phycocyanin‐rich cyanobacteria. Following the decommissioning of Envisat, a data gap occurred from 2012 to 2016 before the launch of Sentinel‐3A (OLCI) in February 2016. OLCI provides continuity with MERIS, offering 300‐m spatial resolution, 21 spectral bands (400–1,020 nm) with enhanced signal‐to‐noise ratios compared to MERIS, red‐edge bands, and near‐daily temporal coverage from its twin satellites Sentinel‐3A and 3B, after the launch of Sentinel‐3B in 2018 (European Space Agency, 2025). The CyAN algorithm harmonizes MERIS and OLCI data using a consistent CI_cyano_ computation and quality control approach to generate long‐term time series of cyanobacteria abundance (NASA Ocean Biology Processing Group, 2025). Together, these sensors enable assessment of cyanobacteria dynamics over two decades at scales relevant to inland lake management, with CI_cyano_ units directly comparable across missions following the harmonization procedure described by Wynne et al. (2021). The year 2012 was excluded from the analysis due to the limited number of images available, with data ceasing in April. This meant that the summer bloom season would be missed, introducing a temporal bias into the statistics.

Quality control flags to indicate potential contamination from clouds, sun glint, shadows, land adjacency effects are applied to the CyAN products (Schaeffer et al., 2022; Urquhart & Schaeffer, 2020; Wynne et al., 2018). Lake shapefiles created for the MERIS/OLCI sensors, intended for CyAN data (Urquhart & Schaeffer, 2020) by having at minimum three water pixels after land removal (Schaeffer et al., 2022), were used as the boundaries for each lake. CyAN SRS data were converted to cyanobacteria abundance and extracted for each lake following Equations 1 and 2. We used daily data to best match the DWR sampling data.

(1) $${\text{CI}}_{\text{cyano}}=10\left(3.0/\left(250.0\;*\;\text{Digital}\;\text{Number}\right)-4.2\right)$$

(2) $$\text{Cyanobacteria}\;\text{Abundance}\;\text{cells}/\text{mL}={\text{CI}}_{\text{cyano}}\;*\;1E+08$$

### 2.4 Using Closest Pixels for SRS Cyanotoxin Approximation

In this study, we are interested in assessing potential bloom spatial variability within other regions of the lake not captured by single SRS pixel estimations or in situ grab samples. In Lopez Barreto et al. (2024), one SRS‐pixel based on the closest water sample location from their field work was used to compare to the closest DWR cyanotoxin collected sample. This was acceptable as that pixel was located within lake limits and not along the lake edge where mixed pixel effects could impact SRS values. Georeferenced points that can be confirmed to be within the lakes edges during fluctuating lake levels were unavailable. To mitigate this, we used a 600‐m buffer around collection sample locations. This distance was chosen because with OLCI's pixel resolution of 300‐m, this would allow up to two pixels of data from the DWR sampling point. If only one pixel was used, there is a higher likelihood of being invalid during dry/low lake level locations. A conceptual diagram of the data extraction can be found in Figure S1 of Supporting Information S1. The average was calculated from the buffer to create a point‐based comparison against the DWR cyanotoxins.

### 2.5 Lake‐Wide Summarization and Data Extraction for the Time Series

To use SRS's capability of measuring entire lakes, especially for those with no in situ sampling locations, we summarized the cyanobacteria abundance across the lakes to a single value by using the lake's daily median cyanobacterial abundance. This single value still captures potential spatial variability of cyanobacteria in the sites rather than single‐point samples.

### 2.6 Point‐Based Comparisons and Lake‐Wide Summarization

To assess cyanobacterial bloom variability within lakes beyond single point‐based samples, we implemented two approaches. First, to enable closest‐pixel comparison with DWR cyanotoxin measurements, we used a 600‐m buffer around each DWR sample location, capturing up to two 300‐m resolution OLCI pixels. This mitigates issues of potentially invalid pixels due to fluctuating lake levels, avoiding edge effects that may impact SRS values. The average was calculated from the buffer to create a point‐based comparison against the DWR cyanotoxins. Second, to harness the spatially comprehensive capabilities of SRS—particularly for lakes lacking in situ measurements—we summarized each lake's cyanobacterial abundance using the median value across all valid lake pixels (Figure S1 in Supporting Information S1). The lakewide median thus retains the central tendency of the abundance values in the lake, capturing the typical bloom condition of the water body, while being less sensitive to localized extremes.

### 2.7 Comparing DWR Cyanotoxins With Sentinel‐3 OLCI Cyanobacteria Abundance

We used the older WHO 1999 cyanobacteria cell‐count guideline over the more recent WHO 2021 guidelines that is based on chlorophyll‐a, which represents total phytoplankton rather than cyanobacteria specifically (Chorus & Welker, 2021). The WHO 2021 guidelines replaced cyanobacteria cell counts with chlorophyll‐a thresholds to standardize bloom risk assessment across monitoring programs and technologies. While cyanobacteria cell counts directly measure potential toxin‐producing organisms, chlorophyll‐a provides a more practical and scalable proxy for total biomass and early warning of bloom development (Seegers et al., 2021). While both chlorophyll‐a and cyanobacteria abundance are used as proxies for bloom risk, cyanobacteria‐specific metrics directly target the organisms capable of producing cyanotoxins. Therefore, the WHO99 GVs cyanobacteria cell‐count–based guideline values remain the most appropriate framework for interpreting CyAN‐derived data.

The OLCI cyanobacteria abundance for each lake were classified into public health‐relevant categories of alert using the WHO99 GVs (Table 1). WHO99 GVs of moderate or high probability (≥20,000 cells/ml) of adverse health effects were classified as an “alert” and WHO99 GVs of relatively low probability (<20,000 cells/ml) were classified as “no alert.” The alert categories were then compared to the DWR advisory categories (Table 1) for that same day. We set a threshold for cyanobacterial abundance of ∼20,000 cells/ml. Because of the limited digital number range (0–255), the actual threshold was 19,642 cells/ml. Pixels that were at or above this threshold were given a “1”, and those below a “0.” While “caution” is an alert that is important for long‐term cyanotoxin monitoring and may help prepare for future closures, the DWR advises users to not enter the water only under warning and danger levels. The “alert” threshold utilized in this study corresponds to the warning or danger tiers of the current California health advisory system, which are based on specific quantitative toxin concentrations (ug/L) for recreational waters, rather than the WHO 1999 or 2021 guidance.

### 2.8 Contingency Table Analysis Agreement Between DWR and SRS‐Based Advisories

We compared the alerts from DWR cyanotoxins against the alerts from OLCI cyanobacteria abundance classified by the WHO99 GV. For our study, we consider the DWR point sample as the true measurement since in situ measurements are typically used to calibrate remote sensing data. We used a contingency table analysis and calculated the overall agreement (OA), false positive rate (FPR), and false negative rate (FNR) to compare SRS alerts and DWR advisories. OA is the sum of the true positive (TP) and true negative (TN) of observations that agree on the level of advisory from both methods (i.e., both SRS and DWR alert trigger) divided by all observations (Equation 3). A false positive (FP) or a false negative (FN) would be the result of a mismatched alert or advisory. The FPR (Equation 4) is the probability that a false alarm would be raised, which for this study means that an alert by SRS would be triggered when there is no alert by the DWR. The FNR, often known as the miss rate, is the probability that SRS would not trigger an alert while the DWR would (Equation 5). A higher FNR would mean SRS‐cyanobacteria underpredicts the DWR advisories, while a high FPR means it overpredicts.

(3) $$\text{Overall}\;\text{Agreement}\;\left(\text{OA}\right)=\frac{\text{TP}+\text{TN}}{\text{TP}+\text{FP}+\text{TN}+\text{FN}}$$

(4) $$\text{False}\;\text{Positive}\;\text{Rate}\;\left(\text{FPR}\right)=\frac{\text{FP}}{\text{FP}+\text{TN}}$$

(5) $$\text{False}\;\text{Negative}\;\text{Rate}\;\left(\text{FNR}\right)=\frac{\text{FN}}{\text{FN}+\text{TP}}$$

We also calculated sensitivity (SN), specificity (SP) and balanced accuracy (BA). SN (Equation 6), or the true positive rate, measures the proportion of actual positives correctly identified. SP (Equation 7), or the true negative rate, assesses the proportion of actual negatives correctly identified. BA (Equation 8), the average of SN and SP, provides a comprehensive measure for imbalanced classes since the total alert rate of the DWR is not evenly split.

(6) $$\text{Sensitivity}\;\left(\text{SN}\right)=\frac{\text{TP}}{\text{TP}+\text{FN}}$$

(7) $$\text{Specificity}\;\left(\text{SP}\right)=\frac{\text{TN}}{\text{TN}+\text{FP}}$$

(8) $$\text{Balanced}\;\text{Accuracy}\;\left(\text{BA}\right)=\frac{\text{SN}+\text{SP}}{2}$$

### 2.9 WHO99 Alert Frequency Maps

Using the OLCI data (2016–2023), we created maps of pixels that were classified as potential cyanotoxin “alerts” following the WHO99 GV (Table 1) to visualize the frequency of alerts on a pixel‐by‐pixel basis across each lake (Equation 9) along with the DWR sampling locations. Each pixel in the map represents the frequency with which that location exceeded the satellite‐derived risk threshold.

(9) $$\text{Pixel}\;\text{Alert}\;\text{Frequency}\;\left(\%\right)=\frac{n\;\text{of}\;\text{pixels}\;\text{above}\;\text{the}\;\text{WHO}99\;\text{GV}\;}{n\;\text{of}\;\text{total}\;\text{images}}\;*100$$

### 2.10 Applying the High‐Risk WHO99 GVs Statewide

We found the bloom frequency, start/end, and the duration (Day of Year end—Day of Year start) of high‐risk bloom dates from 2002 to 2011 and 2016 to 2023 for a total of 76 lakes and reservoirs in California for each year, including the five monitored by the DWR. The lake median CI_cyano_ was converted to cyanobacteria abundance and classified as “high‐risk” and “no alert” following the WHO99 GV of high adverse health effects (≥100,000 cyanobacterial cells/ml) (Table 1). MERIS and OLCI data have differing total days of observations, where OLCI has almost twice as much data per year than MERIS. Even within the OLCI time series, overpass frequency nearly doubled after the launch of Sentinel‐3B in 2018. Change assessments characterized by inconsistent overpass frequency can bias results (Coffer et al., 2026), therefore, we mitigated these overpass differences by calculating the bloom frequency percentage by days of high‐risk divided by the total number of observations per year.

We excluded certain lakes in our analysis such as Lake Tahoe, Salton Sea, Mono Lake and those that are forebays. Lake Tahoe was omitted due to its extremely clear, dark waters (Wang et al., 2020) particularly in the nearshore (Pearson & Huntington, 2019). The Salton Sea and Mono Lake were removed due to their extensive and complicated hydrology and water quality history (Cohen, 2009; Holdren & Montaño, 2002; Stine, 1991; Wiens et al., 1993), which make SRS of cyanoHABs less reliable for those water bodies. We removed the forebays of reservoirs used in the study because of their often stagnant and slower flow rates, which may not reflect overall typical bloom dynamics of typical reservoirs.

## 3 Results

### 3.1 DWR and SRS Agreement Results: Point‐Based Comparison

Rates of OA between SRS‐based advisories and DWR‐based advisories using a point‐based comparison from the DWR sampling site ranged between 56%–100% and the BA was 46%–100% (Table 2). When all samples for all lakes were pooled, OA was 72%, FPR was 27%, FNR was 36%, SN was 64%, SP was 97% and BA was 80%. A minority of the DWR sampling (17%) led to alert level advisories. Lake Oroville had a low sample size, however had an OA and BA of 100%. There were no alerts set by the DWR and SRS at Lake Oroville leading to the 0% SN.

**Table 2:** The Total Dates of Comparison (*n*) and the Results of Overall Agreement (OA), False Positive (FPR), False Negative Rates (FNR), Sensitivity (SN), Specificity (SP), and Balanced Accuracy (BA) of Cyanotoxin Advisories Set by the California DWR Against WHO99 GV Using SRS of OLCI for Each Lake

Name	n	OA (%)	FPR (%)	FNR (%)	SN (%)	SP (%)	BA (%)	DWR alert (%)
Lake Oroville	11	100	0	0	–	100	100	0
San Luis Reservoir	113	84	15	18	82	85	84	35
Castaic Lake	56	88	9	100	0	91	46	3.6
Pyramid Lake	218	56	48	13	87	52	69	11
Perris Reservoir	164	77	11	73	27	89	58	18
All Lakes	562	72	36	53	64	97	80	17

*Note.* The percentage of DWR samples that triggered an alert and the totals for each contingency analysis are shown.

Following Oroville, Castaic had the next highest OA, a low FPR/high SP but a FNR of 100% and SN of 0%. There were no alerts by the DWR for this lake, however SRS created five alerts leading to the high FNR/low SN. Perris followed (OA = 77%) but had the second lowest BA due to the low FPR/high SP, and high FNR/low SN. Over 80% of the samples from Perris Reservoir were no alerts according to the DWR, however the few times it was an alert, SRS under‐detected the alert. Pyramid Lake had the greatest sampling size but had the lowest OA across all lakes. It had the highest FPR across all the lakes but conversely had one of the lowest FNR which is why the BA was higher than two other lakes with a greater OA. Only 11% of the DWR samples were an alert level of advisory, however SRS greatly overpredicted the presented alerts.

### 3.2 DWR and Sentinel‐3 OLCI Agreement Results: Lake‐Wide Comparison

The OA between SRS‐based advisories and DWR‐based advisories using the lake‐wide approach ranged between 54%–100% and BA was 49%–79% (Table 3). The lake‐wide method provided 200 additional samples over the point‐based comparison because there were days that the reservoir water level was too low to produce the given DWR coordinate to extract from. When all samples were pooled, OA was only slightly lower compared to the point‐based approach, but BA deceased by 16%. The highest OA was in Lake Oroville (100%), and the lowest OA was Pyramid Lake (54%). Castaic Lake was the only site to have a higher OA using lake‐wide summaries versus point‐based comparisons. Overall agreement (OA) decreased by 2% at Pyramid Lake and by 11% at San Luis Reservoir when using the lake‐wide approach compared to the point‐based comparison. Castaic Lake was the only site to have an increase for BA using this approach, while Lake Oroville was unchanged and the remaining decreased.

**Table 3:** The Total Dates of Comparison (*n*) and the Results of Overall Agreement (OA), False Positive (FPR), False Negative Rates (FNR), Sensitivity (SN), Specificity (SP), and Balanced Accuracy (BA) Summarized Lake‐Wide

Name	n	OA (%)	FPR (%)	FNR (%)	SN (%)	SP (%)	BA (%)	DWR alert (%)
Lake Oroville	26	100	0	0	–	100	–	0
San Luis Reservoir	146	73	31	17	83	69	76	32
Castaic Lake	124	90	9	33	67	91	79	4
Pyramid Lake	312	54	49	15	85	51	68	8
Perris Reservoir	195	72	13	90	10	87	49	20
All Lakes	803	69	30	42	58	70	64	15

The remaining performance metrics had differing performances across most lakes. The FPR remained stable for all lakes, with only changes up to 2%, except for San Luis Reservoir. The SN and SP decreased by 6% and 27% respectively for all lakes pooled. Pyramid Lake and San Luis Reservoir FNR were consistent. Castaic Lake's FNR vastly improved but was the only instance. Perris Reservoir's FNR increased by 17%, despite DWR alerts slightly increasing. SP for Castaic Lake, Lake Oroville. Perris Reservoir and Pyramid Lake remained stable, while San Luis Reservoir decreased by 16%. Castaic Lake was the only site to have SN increase using a lake‐wide summary, San Luis Reservoir remained stable, and the remaining lakes all decreased.

**Figure 2:**
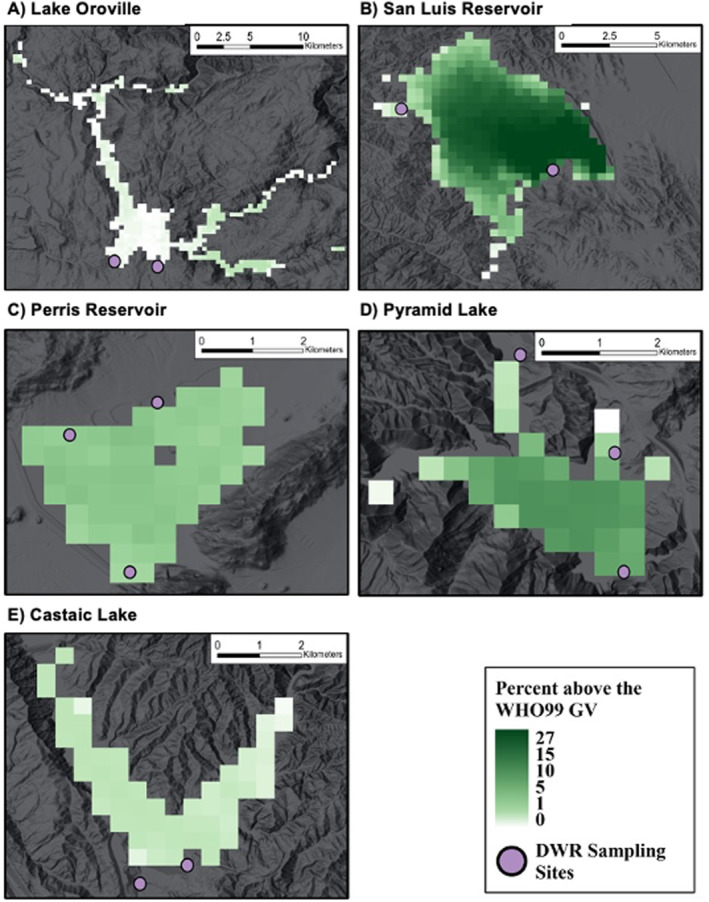
Frequency of alerts following WHO99GV from 2016 to 2023 for each study site. The DWR surface and 1‐m sampling locations are also shown.

There are differing quantities in the total observation days (*n*) presented in Tables 2 and 3, primarily driven by fluctuating lake levels that compromise the SRS extraction. Figure 2 illustrates the reported fixed‐point sampling locations used by the DWR where during drought conditions or other varying lake level conditions, leads to the shift of water collection, moving beyond the fixed coordinates. As DWR does not record the exact GPS location for these adjusted samples, the 600‐m buffer applied to the fixed points for SRS data extraction fails to capture all overpass comparison days. The lake‐wide analyses (Table 3) remain unaffected by this issue, as they aggregate all available remote sensing pixels across the entire water body.

### 3.3 Alert Frequency Spatial Distribution Using the WHO99 GV

The alert frequency maps (Figure 2) provide a spatial visualization of all cyanobacteria classified to WHO99 GV alerts from 2016 to 2023. The values represent the portion of observations in the SRS time series that were classified as an alert.

The alert frequency values across all lakes range from 0% to 27%. San Luis Reservoir had the highest frequency of WHO99 GV alerts in the time series, with a maximum of 27% of the time occurring at various locations within the lake. Other sites with a higher alert frequency were Perris Reservoir (maximum = 6.7%) and Pyramid Lake (maximum = 11.9%). The lakes with the overall lowest frequency of alerts were Castaic Lake (maximum = 1.5%) and Lake Oroville (maximum = 2%).

Castaic Lake and Perris Reservoir appear to have a spatially homogeneous distribution of cyanobacterial abundance above the WHO99 GV (Figure 2). San Luis Reservoir has the highest frequency of alerts and spatially, these instances do not occur in bays or other small clusters of areas with high values, but rather high frequency that is distributed throughout the lake. The greatest values appear to be on the east side of the lake. Pyramid Lake appears to have denser alert rates toward the center of the lake and have lower values around the edges, which may be driving the lower OA values calculated for this site because the DWR sampling sites were all near the lake edge.

### 3.4 Cyanobacteria Toxin Alerts Across California Lakes and Reservoirs

Most lakes in our study from California are found in the north (59% of all lakes), 26% in central, and 15% in the south (Figure 1). Of the 10 lakes with the greatest rates of alerts, six were in southern California, three in central, and one in northern California (Table 4).

**Table 4:** Top 10 Lakes in California With Frequently Occurring WHO99 GV Alerts (High‐Risk) as Estimated From SRS Data From 2002 to 2011 and 2016 to 2023, Where 9 Are Not Owned, Managed, or Sampled by the DWR

Lake/Reservoir name	Frequency above WHO99	Region
Lake Elsinore	87.8	Southern
Lake Crowley	25.3	Central
Big Bear Lake	21.4	Southern
Sweetwater Reservoir	18.1	Southern
Clear Lake	17.2	Northern
San Luis Reservoir	14.7	Central
Calaveras Reservoir	13.9	Central
Lake San Antonio	13.2	Central
Diamond Valley Lake	13.0	Southern
Lake Henshaw	12.0	Southern

The 10 lakes with the highest frequency of alerts had a range of 12.0%–87.8% across both time periods of 2002 to 2011 and 2016 to 2023 according to SRS (Table 4). There were 16 lakes which had 0%, 46 lakes had a range of 0.05%–10% and 10 lakes had a range of 10%–15% of alerts above the WHO99GV. The remaining four lakes were above the WHO99 GV 15% of the time, where one site had a near 100%. The complete table of our statewide analysis can be found in Table S3.

SRS provides daily data for cyanobacteria (assuming there is no cloud or image disruptions), while there are limited cyanotoxin data available on the California State Water Board website (https://mywaterquality.ca.gov/habs/where/freshwater_events.html) for the 10 lakes. Clear Lake had the most data publicly available, over three times than the second greatest, while Calaveras Reservoir had no data available.

### 3.5 Temporal Shifts in High‐Risk Algal Blooms Across California

**Figure 3:**
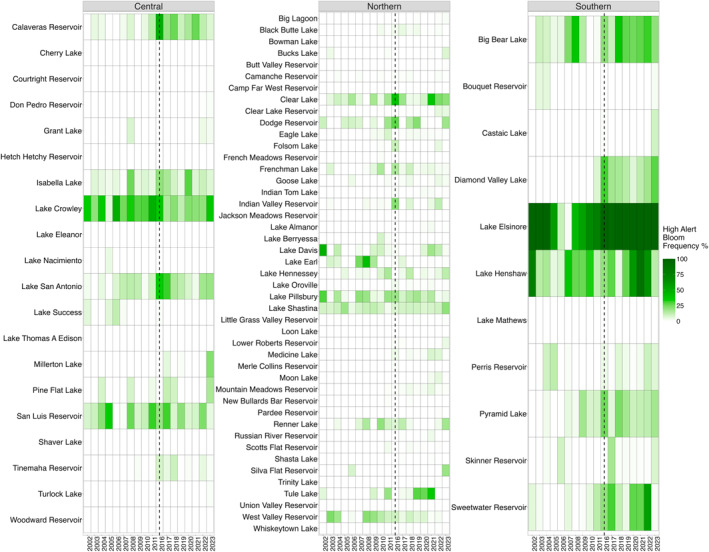
Annual percentage of observations classified as high‐risk based on the WHO99 GV, normalized by the total number of valid observations per year to account for differences in sensor revisit frequency and data availability. The vertical dotted line denotes the 4‐year data gap between satellite missions.

While numerous water bodies exhibit minimal to no high‐alert bloom days, there are lakes such as Lake Elsinore, Lake Henshaw and Big Bear Lake in the southern region, and intermittently Lake Crowley and San Luis Reservoir in the central, that show significant and often intense bloom activity. Multiple lakes that did not experience a bloom from 2002 to 2011 (total of 33), turns to experience some bloom activity in 2016–2023. The total number of lakes that never experience a bloom across the study are 16 (Figure 3).

**Figure 4:**
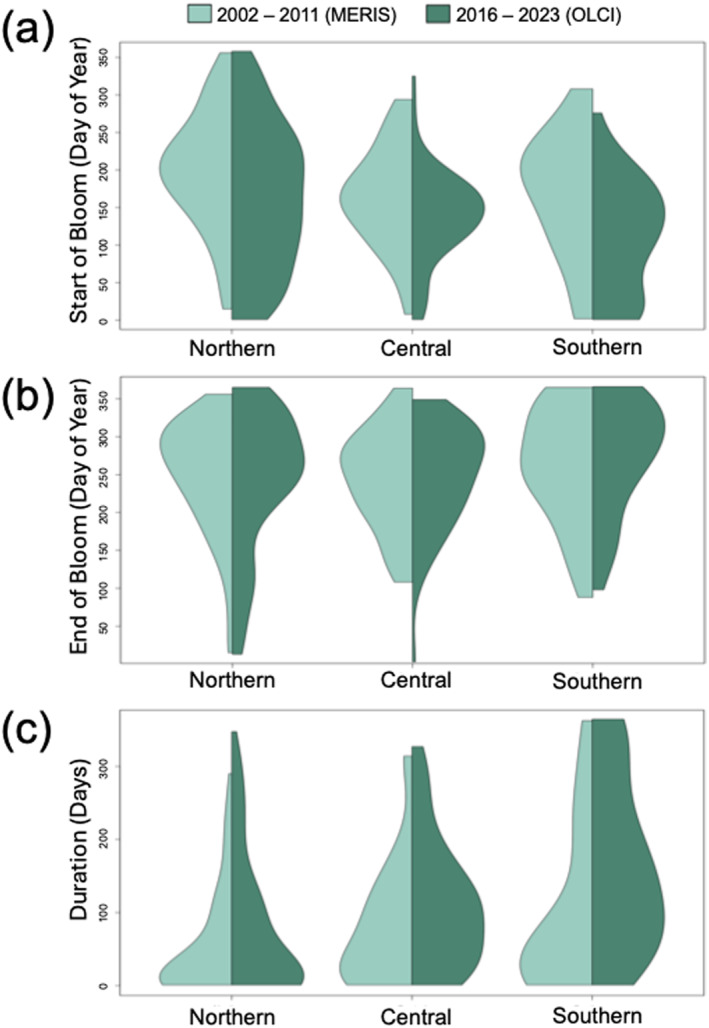
Violin plots of the start (a) and end (b) dates of high‐risk bloom and their duration (c), calculated as the end of bloom day of year minus the start of bloom day of year, across California for 2002–2011 (using MERIS) and 2016 to 2023 (using OLCI).

Non‐parametric statistical comparisons using a two‐tailed Wilcoxon rank‐sum test (*p* < 0.05) revealed significant temporal shifts in SRS alerts between the early (2002–2011) and later (2016–2023) time periods across all examined regions. For our statistical analysis, we removed 2012 from the earlier period due to minimal data (Figure 3). In the central region, we observed longer duration of alerts (approximately 32 days) in 2016–2023 compared to 2002 to 2011 (Figure 4). These changes were accompanied by an earlier onset (27 days) and a later end of high‐risk days (5 days). The northern region exhibited a similar trend, with alert duration increasing by 27 days and alerts starting 35 days earlier, however ending 8 days earlier (Figure 4). The southern region showed the most pronounced changes in the later period, with alert duration extending by 73 days. Alerts in southern California initiated 58 days earlier and concluded approximately 15 days later (Figure 4). When we excluded Lake Elsinore from the southern region analysis, alert duration was 94 days, blooms started 75 days earlier and lasted 19 days later. These findings demonstrate more frequent, longer and earlier high‐risk bloom periods across all regions.

We found that in central California, the bloom frequency average for the early period was 3.5% and became 5.3% in the later period, northern California's early period average was 1.5% and was 2.6% in the later period and southern California had an average 9% bloom frequency in the early period and more than doubled to 20.5% in the later period. By removing Lake Elsinore from the southern region, the earlier period bloom frequency was 2.7% and the later was 10.7%.

## 4 Discussion

### 4.1 Point‐Based Versus Lake‐Wide Against DWR Cyanotoxins

One of the motivations of this study is to use a metric that summarizes the conditions across a lake effectively. Comparing an alert that was classified using the lake‐wide median against the DWR samples is comparing a summarized value from multiple locations to a point sample. Because of spatial differences, it is reasonable that the data closest to the validation site would have a greater overall agreement against a value that condenses cyanobacteria spatial variability to a single value.

Using a point‐based comparison outperformed a lake‐wide according to the BA. The OA treats correct predictions and errors as equally important, whereas BA offers a more nuanced perspective by focusing specifically on true positives and true negatives. There were over six‐times more DWR non‐alerts than alerts, which could overestimate the true accuracy of using SRS for cyanotoxin monitoring. The BA helps interpret these unequal classes. We assumed that the lake‐wide results would be similar to the point–based results, which was true, but we also expected them to have a lower agreement rate due to the nature of summarizing a large area to compare to a specific location. With the slightly lower OA using lake‐wide summaries, we can conclude that it does not perform as well as a point‐based comparison, however the BA had a greater decrease.

Contributing factors to the high FNR and false positive rate (FPR) include the concentration of algal blooms at lake edges, minimal DWR alerts relative to non‐alerts, and the inability of SRS to explicitly detect cyanotoxins. The FNR was 42% for pooled samples using the lake‐wide approach, which is 6% higher than using the point‐based approach. However, there were a total of 147 DWR alerts (15%) across all sites compared to the 683 non‐alerts (85%). The quantity of alerts is not necessarily a small sample size, however distributed across five sites may not be enough to confidently state this high of an FNR. Another highly plausible explanation is that the cyanobacteria found in the lake may not be toxin‐forming (O’Neil et al., 2012; Stumpf et al., 2016). SRS captures cyanobacteria by detecting the associated pigments (phycocyanin) but cannot explicitly sense cyanotoxins because these harmful substances do not have distinct spectral signatures detectable by current remote sensing technologies.

Using a lake‐wide approach in a lake well known for its persistent and higher advisory levels may be more appropriate than using point‐based collection/measurements. San Luis Reservoir has the greatest rate of triggering an alert (Tables 2 and 3) compared to the other four DWR sampled lakes. SRS‐based alerts are found throughout the lake, however, appear the lowest around the lake's edges (Figure 2). Although DWR's near‐shore sampling informs recreational users near the lake's edge, our interest lies in assessing the toxic potential of the entire lake, given that recreational activities such as boating, fishing, and swimming often take place further from the shore. Using a point from one of the lake edges may underestimate the toxicity of the lake if there are denser blooms away further within the lake. Lake Oroville on the other hand has overall low cyanobacteria alert values (Figure 2). This tracks with the outcomes of Table 2 where all DWR samples cyanotoxins did not trigger an alert, however, there may be locations in the lakes where alert level cyanoHABs may have occurred but were not being sampled by the DWR (Figure 2). Castaic Lake and Perris Reservoir spatially homogeneous appearance (Figure 2) may be the result from high winds or other horizontal mixing processes. Frequent and high winds (Table S1 in Supporting Information S1) may influence bloom location, where when averaged over time, could lead to similar frequencies across the lake which would be missed if only relying on select point samples.

Cyanotoxin production in algal blooms is highly variable and depends on specific environmental conditions (Chorus & Bartram, 1999; Davis et al., 2009), meaning that cyanobacteria found in different areas of the same lake may not always produce toxins (Szlag et al., 2015). According to the DWR data, not all lakes are toxic such as Lake Oroville or (typically) Castaic Lake. While these lakes have little to no cyanotoxins found when sampled, the SRS data also align with these findings by indicating low recommended alerts during the sampling period. But there may be differing quantities or types of cyanobacteria occurring in the lake that may be different in the locations collected from the DWR. In Figure 2, Lake Oroville's DWR sampling points are in the lowest areas for alerts, while the eastern side has greater frequency rates which may indicate larger cyanobacteria concentrations compared to current sampling. SRS can help water managers by providing detailed spatial and temporal data on algal bloom distributions, highlighting areas with high concentrations of cyanobacteria for targeted cyanotoxin testing.

San Luis Reservoir would be an ideal site to use SRS data to help monitoring efforts because of its well documented toxicity and bloom spatial variability (Figure 2). Basalt Boat Launch (Figure 2b; south‐west point) has over three times the data (*n* = 150) and double the alert rate (34%) that Dinosaur Point (Figure 2a; north‐east point) has (*n* = 41 and alerts = 15%), which is where current and future cyanotoxin monitoring is being done because of road closures. This aligns with the pattern shown in the alert frequency maps (Figure 2), where areas close to Basalt Boat Launch have trends of higher WHO99 GV alerts, while the Dinosaur Point site has some of the lowest rates of the figure. Since we are attempting to summarize the cyanotoxin potential of the whole lake, the Dinosaur Point location may be underestimating the bloom status of further areas at San Luis Reservoir that can be found in the Basalt area.

### 4.2 Lakes and Reservoirs With High Cyanotoxin Risk

The analysis of high‐risk bloom dynamics across California revealed that the top 10 lakes with the greatest lake‐wide alert frequency were primarily found in southern California, followed by central California, and finally northern California. These findings are complementary to the work of Urquhart et al. (2017), who utilized the same satellite sensor, MERIS. The study focused on changes in total bloom area (spatial extent) from 2008 to 2011, finding that the greatest spatial extent increases were in central California, followed by southern California, and a minimal increase in northern California. Our study researched high‐risk event frequency and duration as defined by the WHO GVs and offer a more direct proxy for public health exposure risk.

Northern California is colder and has higher rates of precipitation, central California has a mix of climates with hot summers, and southern California is warmer and drier. With limited precipitation, if lake levels become low, then entering nutrients could become more concentrated compared to a fuller lake (Brasil et al., 2016; Jeppesen et al., 2015; Özen et al., 2010). Southern California, especially closer to the coast, has greater urban cover (U.S Census Bureau, 2020) which may influence the concentration, rate and type of nutrients entering the system (Müller et al., 2019). While central California may not be as urban, it is still well known for its hot temperatures and dominant agriculture which would also support high nutrient carrying runoff (Moss et al., 2011). Hotter temperatures and higher concentration of nutrients would greatly support algal growth which would explain our findings.

Our analysis reveals that there are more frequent high‐risk algal bloom events across various California lakes and reservoirs. Both statistical comparisons and visual representation of the data indicate more frequent high‐risk bloom periods, particularly in recent years. This pattern is observed across all three regions (central, northern, and southern California), although the southern region exhibits the most pronounced changes, with the highest bloom durations and number of high‐risk bloom days. While there is spatial variability in the extent of these changes, the overall trend suggests a statewide intensification of harmful algal bloom activity, highlighting the need for increased monitoring and management efforts to mitigate the potential ecological and public health impacts.

The lake with the highest WHO99 GVs frequency was Lake Elsinore, which has a documented history of HABs which is likely why it has one of the highest data samples available. The Santa Ana Regional Water Quality Control Board samples various points around the lake and have a similar procedure as the DWR for their cyanotoxin monitoring (City of Lake Elsinore, 2024). Clear Lake has a similar monitoring plan in place (Big Valley Band of Pomo Indians, 2024). Lake Henshaw's agency states that they collect cyanotoxins weekly from two sites in the lake. The other lakes had some data available in the portal, except Calaveras Reservoir, where there was no cyanotoxin data readily available online at the time of publication. The monitoring schedule for Lake Crowley and Diamond Valley Lake was not readily available, however there have been previous news reports of danger warnings of detected cyanotoxins/HABs (Comstock, 2024; Williams, 2023; Green, 2018).

Knowing where there are confirmed high rates of cyanobacteria (Figure 2), and paired with cyanotoxin data (Table 4), can significantly enhance future cyanotoxin research. Having a list of lakes most affected by cyanobacterial blooms can be used to prioritize monitoring efforts and allocate resources more efficiently to these regions. Combining SRS cyanobacteria and in situ cyanotoxin data, researchers can further improve the accuracy of bloom assessment and better understand the correlation between cyanobacteria presence and toxin production, improving predictive models and risk assessments.

### 4.3 Closing Spatial and Temporal Data Gaps

SRS provides substantially more spatial cover estimates of cyanobacteria compared to cyanotoxin samples collected by various agencies or managers. SRS allows for standardized cyanobacteria retrieval with a higher revisit frequency, and the data are freely accessible the following day through CyAN (https://oceancolor.gsfc.nasa.gov/about/projects/cyan/). In contrast, in situ cyanotoxin data are distributed across different agencies, potentially using varying sample collection methods, and may not be publicly available or shared promptly. DWR's initial cyanotoxin sampling season is influenced by local reports and the duration of detected toxicity levels at sampling locations. SRS provides near real‐time monitoring and trends of cyanobacterial bloom development, helping water managers determine the optimal timing for cyanotoxin testing during peak algal activity periods. Including SRS data in current monitoring plans can enhance the focus on both timing and location of potential cyanotoxin hotspots for targeted testing.

Pyramid Lake had a low FNR, meaning that SRS has a good rate of detection when it is potentially toxic. However, it had the lowest OA because it often overpredicts cyanotoxin risk. While this site's DWR alert frequency is relatively low (7.9%), presence of higher toxicity levels is not constant throughout the time series. In 2021, there were 19 DWR alerts compared to the previous and following year of only three. In 2023 there were also only three DWR alerts, however the levels of cyanotoxins prompted wide news coverage (Rodriguez, 2023) and the DWR urged visitors to not enter the lake (DWR, 2023). This means that cyanobacteria in Pyramid Lake have confirmed cyanotoxins, however the sampling location may not be reflecting potential hotspots which appear to be the center of the lake according to Figure 2, or the algal bloom intensity may not be consistent over the time‐period. Pyramid Lake has three official sampling locations, but our analysis of pixel location frequency (Figure 2) shows the highest SRS alerts are found across the wider lake body, while two out of the three grab sample locations are at lower‐risk locations, which may explain the high FNR. If a bloom is concentrated in the main body of the lake, where the SRS detects high risk, but the DWR grab sample is collected in a localized area with less bloom activity, the result is a false positive for the SRS data point. This highlights the inherent limitation of comparing a single grab sample's concentration to a spatially extensive satellite image. Having more spatially varied sampling might be able to address the poor OA at Pyramid Lake.

While the paper focuses on recreational waters, the presented methodology for assessing cyanobacteria abundance could be adapted to monitor source water quality. The ability of SRS to provide consistent, near real‐time data on bloom patterns could be particularly valuable for managing lakes that serve as drinking water reservoirs. Early detection of potential bloom events near water intakes would allow for proactive measures by water treatment facilities, such as adjusting treatment processes or implementing alternative water sources, thereby minimizing the risk of cyanotoxins impacting drinking water supplies. The broad spatial coverage of SRS could also aid in identifying regional bloom events that might affect multiple water sources, facilitating coordinated management strategies.

### 4.4 Interpreting Discrepancies in Satellite‐Derived Public Health Risks

An important consideration when interpreting the comparison between SRS data and in situ results is the difference between cyanobacterial biomass and cyanotoxin concentration. SRS detects the presence and extent of the bloom using proxies for pigment concentration (biomass), while public health risk is determined by the concentration of toxins (e.g., Microcystin) in the water. A lack of agreement between SRS and cyanotoxin measurements is expected because toxin production is not strictly correlated with cell abundance (Mishra et al., 2021). Consequently, toxin concentrations can be present even when the bloom biomass is sparse. The comparison between SRS‐derived bloom estimates and in situ data is further complicated by the limitations in toxin measurement. The DWR in situ data relies on the detection of a limited toxins (Microcystins, Anatoxins, and Saxitoxins) measured by methods such as ELISA. We acknowledge that the absence of Microcystin detection does not imply the absence of all cyanotoxins, as other species classes may be present or may be below the detection limit of the method used. The discrepancy is rooted in the comparison of SRS cyanobacteria abundance risk indicator against a specific, and often temporally and spatially constrained, toxin measurement also shown in Whitman et al. (2022).

Given this difference, managers should use the SRS data set as an early warning trigger for enhanced surveillance. When a high SRS signal is observed but in situ toxin levels remain low, the management response should involve increased sampling frequency and/or expanded toxin analysis to test for other toxin classes or impending toxin release. Conversely, instances of low SRS alerts but high in situ toxin require the continuation of fixed‐point, high‐frequency monitoring as the primary safety measure. We do not advice SRS as a replacement for in situ analysis, but as an important extension of large‐scale screening. This reinforces the role of SRS as an effective large‐scale screening tool for identifying areas of high risk, which must then be followed up by targeted, in situ analysis to determine the precise level of public health hazard.

### 4.5 Study Limitations & Future Research Priorities

California was in a period of drought in 2007–2009, 2012 to 2016, and 2020 to 2022, while the years 2005, 2010 to 2011, and 2016 to 2019, and 2023 were considered exceptionally wet (California DWR, 2023) During drought, reduced water flow, increased water residence time, and heightened temperatures create ideal, stagnant conditions for cyanobacterial growth. This effect is often exacerbated by concentrated nutrients resulting from evaporation and limited dilution (Gámez et al., 2019; Lehman et al., 2017). Conversely, wet years, which often align with El Niño phases, can trigger bloom events through increased runoff and high nutrient loading from rainfall and snowmelt. However, while an increase of nutrient presence promotes algal growth, high flow rates can also dilute and flush out bloom biomass and nutrients, which may temporarily reduce blooms (Michalak, 2016; Reichwaldt & Ghadouani, 2012). This cycle of severe droughts and wet years may explain the large inter‐annual variance observed in our SRS record.

A disadvantage of using SRS for cyanobacteria detection is the lower spatial resolution, particularly in the case of Envisat and Sentinel‐3, potentially missing small‐scale blooms and providing less detailed highly local information compared to in situ data and other finer resolution satellites such as Sentinel‐2 (10–20‐m resolution). Additionally, SRS can be affected by atmospheric conditions such as cloudy weather, which can obstruct accurate data collection. The ideal SRS data set is not yet available, but future plans for a satellite with high spatial and suitable spectral resolution are underway with NASA's Surface Biology and Geology (SBG) mission.

The WHO99GVs are only applicable to lakes that have confirmed cyanotoxins found in the system. SRS can accurately detect cyanobacteria biomass presence and levels, however using the WHO99GV, we are assuming denser blooms are toxin‐producing, which is not always true. Detected cyanobacteria presence does not ensure cyanotoxin exposure because not all cyanobacteria strains produce toxins (Bláha et al., 2009; Chorus, 2001; Chorus & Bartram, 1999; Szlag et al., 2015). The production of cyanotoxins is influenced by various environmental factors that can vary by site (Boopathi & Ki, 2014; Holland & Kinnear, 2013; Kaebernick & Neilan, 2001; Neilan et al., 2013). While cyanobacteria presence can signal potential risks, actual toxin levels must be measured directly to confirm exposure and assess health risks (Stumpf et al., 2016).

The observed statewide trend towards longer and earlier high‐risk algal bloom periods (Figure 3) likely reflects a confluence of factors, including regional climate variations and nutrient loading within each watershed. The higher rates in the southern region, even when excluding the highly impacted Lake Elsinore, suggest potentially distinct regional drivers compared to northern California. While this analysis highlights the significant temporal and spatial patterns of bloom intensification, future research should aim to investigate the specific contributions of source water characteristics and other localized environmental factors to the bloom dynamics observed in individual lakes and reservoirs. Understanding these localized influences will be crucial for developing targeted and effective management strategies.

The comparison of the two periods reveals more frequent and higher rates of high‐risk bloom metrics statewide. However, the interpretation of the differences observed between the 2002 to 2011 (MERIS) and 2016 to 2023 (OLCI) periods requires some caution. Although the algorithms applied were designed to ensure data consistency and inter‐sensor comparability (Urquhart & Schaeffer, 2020; Wynne et al., 2021), it is a known challenge that residual differences in sensor radiometric sensitivity and noise can exist. While annual bloom frequency was normalized by the total number of valid observations to mitigate these effects, interannual differences in data availability may still influence the magnitude of reported trends. While the consistent and widespread nature of the observed intensification strongly suggests a real change in bloom dynamics, the exact magnitude of these high rates must be viewed as an estimate, given by the limitations of combining data from two separate satellite missions.

### 4.6 Additional Sources for Water Quality Data in California

There are other monitoring organizations that share similar and other water quality data like the DWR such as the Klamath Basin Monitoring Program, East Bay Regional Park District, Big Valley Band of Pomo Indians (Clear Lake focused), and Kern County Public Health. Some organizations' frequency, methods for sampling, or time frame are not the same as the DWR's. Many organizations give clear past and current water quality advisories concerning HABs, however the actual cyanotoxin data is not available alongside these notices. Several participating organizations contribute water quality information via CEDEN, the California Environmental Data Exchange Network. CEDEN represents a collaborative initiative across California's water and environmental sectors, welcoming federal, state, county, and private entities eager to share data statewide. Facilitating data exchange among diverse groups, the CEDEN network aims to provide public access to water and environmental data (California Water Code Section 12400, 2016). If those that collect water quality data were to publish their data to this public repository, then this can support further collaboration, transparency and provide further informed decision‐making for important sites.

## 5 Conclusion

This study's demonstration of satellite remote sensing (SRS) for monitoring cyanobacteria and predicting potential cyanotoxin exposure in recreational lakes holds significant implications for safeguarding public health. The observed high‐risk cyanobacteria blooms across all regions of California underscores the importance of monitoring and timely information to inform and safeguard public health.

Our study highlights the effectiveness of point‐based cyanotoxin monitoring over lake‐wide summaries. From the five study lakes where DWR data were available, San Luis and Perris Reservoir produce the greatest rates of cyanotoxins according to the DWR data. The OA of these lakes for both point‐based and lake‐wide approaches yielded reasonable agreements (>70%). Lake Oroville and Castaic Lake had the lowest cyanotoxin alerts and had the highest OA. The nuanced evaluation provided by balanced accuracy (BA) versus overall accuracy (OA) reveals that while lake‐wide approaches offer a broader perspective, they often underestimate the true variability and potential hotspots within a lake. Further investigation should focus on utilizing lake‐wide summaries to compare point samples, such as having varied locations of in situ samples. Overall SRS of cyanobacteria of inland lakes provide reasonable agreement but should have in situ data to help establish a better comprehension of the toxicity rates of the lakes.

The spatial and temporal variability in cyanotoxin presence underscore the need for targeted, high‐resolution monitoring strategies. In regions with higher cyanobacteria risk, such as southern California, the integration of remote sensing data with traditional sampling methods can enhance monitoring precision and resource allocation. By identifying lakes with persistent and high advisory levels, we can prioritize these areas for more intensive study and intervention. The combination of SRS and in situ data collection provides a robust framework for understanding cyanobacteria dynamics and mitigating their impacts.

## Conflict of Interest

The authors declare no conflicts of interest relevant to this study.

## Supporting information

Supporting Information S1

Table S3

## Data Availability

Data and scripts created and used for this paper (with the exception of SRS images) are available in Lopez Barreto (2026) where it is free to access and use with no registration required. SRS image files can be found on NASA's Earthdata Search Engine. The files associated with this data set are licensed under a Creative Commons Attribution 4.0 International license. The list of names of all satellite remote sensing images used are found in our repository.
