# Supplementary material for: Statewide Intensification of Harmful Algal Blooms Across California Lakes and Reservoirs
Source: Geohealth. 2026 Apr 3;10(4):e2025GH001627. doi: 10.1029/2025GH001627 (PMC13052314; doi:10.1029/2025GH001627)
Supplement: Supplementary file 1 — Supporting Information S1 [file GH2-10-e2025GH001627-s001.docx]

*GeoHealth*

Supporting Information for

**Statewide Intensification of Harmful Algal Blooms Across California Lakes and Reservoirs**

Brittany N. Barreto Martinez^1,2^, Erin L. Hestir^2^, Marc W. Beutel^2^, and Christine M. Lee^3^

^1^ Department of Civil Construction, and Environmental Engineering, San Diego State University

San Diego, CA, United States

^2^Department of Civil & Environmental Engineering, University of California Merced

^3^NASA Jet Propulsion Laboratory, California Institute of Technology, Pasadena, CA, United States

**Contents of this file**

Figure S1

Tables S1 to S2

**Additional Supporting Information (Files uploaded separately)**

Captions for Table S3

**Introduction**

In this supporting information, we provide the background summary of climatic, environmental and purpose/use of the reservoirs owned by the California Department of Water Resources which was used in our study. We also provide a conceptual schematic diagram showing how we calculated our point and lakewide satellite cyanobacteria data assessments. We also include a table (uploaded separately) of the bloom frequency and how much data was available for all 76 lakes in our study.


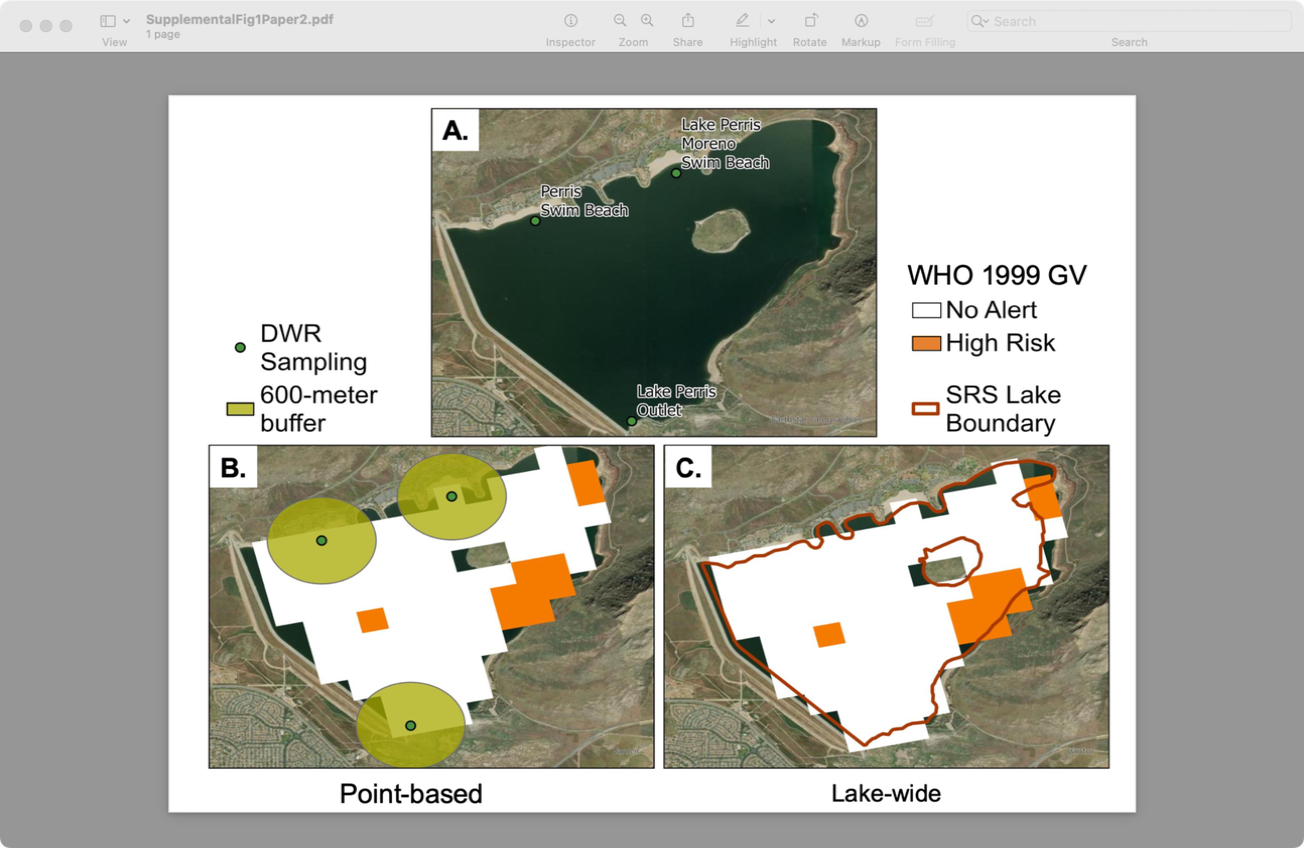


**Figure S1.** Conceptual figure of the comparison of point-based and lake-wide algal to in-situ DWR data. Figure S1A. shows Lake Perris, identifying the three locations where the DWR conducts in-situ sampling (green dots). Figure S1B. depicts the localized comparison method, where a 600-meter buffer is applied around each DWR sampling point where the median was used for comparison. Figure S1C. outlines the SRS lake boundary (red line), where all available water pixels within the boundary are aggregated to calculate a single lake-wide metric (median). In both B and C, the World Health Organization (WHO) 1999 Guidance Values (GV) for risk classification was classified using the SRS CyAN data.

| **Name** | **Trophic Level** | **Area**  **(Acres)** | **Volume (Acre- foot)** | **Mean depth**  **(Meters)** | **Watershed Area (Kilometers^2^)** | **Mean Residence Time (Days)** | **Primary Purpose** | **Other Purposes** | **Surface Elevation (Meters)** |
| --- | --- | --- | --- | --- | --- | --- | --- | --- | --- |
| Lake Oroville | Oligotrophic | 15,805 | 49,587 | 0.96 | 9301 | 377 | Flood risk reduction | Irrigation, recreation, hydroelectricity, and water supply | 274.3 |
| San Luis Reservoir | Eutrophic | 13,000 | 48,139 | 1.2 | 213 | 59,577 | Hydroelectricity | Irrigation. Recreation, and water supply | 165.5 |
| Castaic Lake | Oligotrophic | 2,235 | 27,273 | 3.8 | 410 | 2056 | Water supply | Irrigation and recreation | 467.9 |
| Pyramid Lake | Eutrophic | 1,360 | 4,252 | 0.96 | 744 | 602 | Water supply | Recreation and hydroelectricity | 794.3 |
| Perris Reservoir | Oligotrophic | 2,340 | 10,320 | 1.3 | 22.6 | 17,700 | Water Supply | Irrigation and recreation | 487.7 |

**Table S1.** Summary of the physical and geographic (Meyer et al., 2023; US Army Corps of Engineers National Inventory of Dams, 2024; Messager et al., 2016; CA DWR, 2024) reservoir metrics in this study in order from largest to smallest by area. Trophic level was determined by the most prevalent class for each lake from 2015-2019 by the Meyer et al. (2023) dataset.

| **Name** | **Latitude** | **Longitude** | **Distance to Nearest City (KM)** | **NLCD 2019 \| 2021** | **WHR** | **Wind Speed (MPH)** | **Peak Gust Speed (MPH)** | **Air Temperature (°F)** | **Rain (inches)** |
| --- | --- | --- | --- | --- | --- | --- | --- | --- | --- |
| Lake Oroville | 39.543 | -121.491 | 4.8 | Cultivated Crops | Annual Grassland | 7.84 | 12.1 | 63.4 | 23.3 |
| San Luis Reservoir | 37.033 | -121.133 | N/A | Cultivated Crops | Annual Grassland | 6.44 | 13.2 | 64.8 | 6.09 |
| Castaic Lake | 34.527 | -118.611 | 1.6 | Scrub/Shrub | Mixed Chaparral | 9.46 | 60.1 | 61.1 | 12.8 |
| Pyramid Lake | 34.644 | -118.764 | 19.3 | Scrub/Shrub | Mixed Chaparral | 11.0 | 63.7 | 60.3 | 10.8 |
| Perris Reservoir | 33.858 | -117.183 | 0.6 | Scrub/Shrub | Urban | 4.62 | 52.3 | 65.6 | 6.2 |

**Table S2:** Summary of the geographic coordinates, land characteristics and the mean climate of our study. The primary national land class (NLCD) (U.S Multi-Resolution Land Characteristics Consortium), for both 2019 and 2021, and the California wildlife-habitat relationship (WHR), the vegetation type most important to wildlife (California Department of Forestry and Fire Protection, 2023), was summarized for each lake’s Hydrologic Unit Code-8 sub-basin.

**Table S3:** All satellite resolvable lakes (n = 76) in California used in our study with the total days of WHO99 GV alerts (both moderate and high-risk), total days of SRS data and the bloom frequency of WHO99 GV alerts as estimated from 2002 – 2011 and 2016-2023. This data is uploaded separately.
